# Investigating the prevalence of anxiety and depression during the first COVID‐19 lockdown in the United Kingdom: Systematic review and meta‐analyses

**DOI:** 10.1111/bjc.12360

**Published:** 2022-02-09

**Authors:** Luca Marie Dettmann, Sally Adams, Gemma Taylor

**Affiliations:** ^1^ Addiction and Mental Health Group Department of Psychology University of Bath UK

**Keywords:** anxiety, COVID‐19, depression, lockdown, mental health, United Kingdom

## Abstract

**Background:**

The COVID‐19 pandemic has had a significant impact on mental health. Specifically, the stringent lockdown restrictions have heightened anxiety and depression. Therefore, monitoring and supporting the mental health of the population during these unprecedented times is an immediate priority.

**Methods:**

In this systematic review and meta‐analyses, articles that explored the prevalence of anxiety and depression during the first COVID‐19 lockdown in the United Kingdom were included. We searched the databases Embase, Medline (PubMed), Web of Science, and PsycINFO for cross‐sectional studies. We conducted meta‐analyses of prevalence rates using a random‐effects model, and the heterogeneity of studies was examined using the *I* ^2^ index.

**Results:**

Fourteen studies involving 46,158 participants were included in the review. The studies use clinical cut‐off scores on anxiety and depression measures to define cases. While the prevalence of anxiety was 31.00% (95% CI = 26.00 to 35.00), the prevalence of depression was 32.00% (95% CI = 29.00 to 35.00). The prevalence of anxiety pre‐pandemic was 4.65%, indicating a 26.35% increase. Whereas the prevalence of depression pre‐pandemic was 4.12%, indicating a 27.88% increase. Moreover, participants experienced a slightly greater prevalence of depression than anxiety by 1.00%.

**Conclusions:**

To conclude, the first COVID‐19 lockdown in the United Kingdom increased the prevalence of anxiety and depression among the general population, compared to pre‐pandemic data. Hence, it is vital that policymakers and mental health services maximize their efforts to monitor mental health and provide interventions to support those in need.

**Practitioner points:**

**Clinical implications**

- Awareness of the high prevalence of anxiety and depression during the first lockdown in the United Kingdom can inform policy development that substantial effort, time, and funding of mental health services are required to support those in need.
- Similarly, awareness of the prevalence of anxiety and depression in the United Kingdom can contribute to the development of nation‐specific interventions and initiatives.

**Limitations**

- The current review focuses on the UK general population which does not allow the findings to be generalized to the global population.
- The indirect comparison of the current prevalence rates with the corresponding pre‐pandemic prevalence rates obtained from a different study sample increases individual differences, weakening the reliability of the findings.

## Background

Coronavirus disease 2019 (COVID‐19) is an infectious disease caused by severe acute respiratory syndrome coronavirus 2 (SARS‐CoV‐2). It was discovered in December 2019 and has since proliferated into a global pandemic with almost 250,000,000 cases and almost 5,000,000 deaths according to the World Health Organisation (WHO) (WHO, 2020).

To combat the spread of COVID‐19, national lockdowns have been set in place by governments. Lockdowns reduce social interaction by implementing travel restrictions, curfews, and stay‐at‐home orders; closure of borders, schools, non‐essential shops and production, and public venues (Niedzwiedz et al., 2020); and social distancing and quarantine rules are stipulated. These restrictions have had various social and economic consequences, including potential increases in loneliness, substance use, and domestic abuse (Bhavsar, Kirkpatrick, Calcia, & Howard, 2021; Groarke et al., 2020; Panchal et al., 2020); and the global economy has deteriorated resulting in widespread job loss, reduction in personal income, and people having to work from home (Nicola et al., 2020).

The United Kingdom is among the most affected countries in Europe with over 140,000 deaths (WHO, 2020). Consequently, the first UK lockdown spanned 7 weeks from 23 March to 13 May 2020. Several studies have been conducted to assess the prevalence of anxiety and depression during the first COVID‐19 lockdown in the United Kingdom. Studies estimate that during the first lockdown, between 19.60% (Bu, Mak, & Fancourt, 2021) and 67.51% (White & Van Der Boor, 2020a) of the population experienced anxiety, and between 18.85% [8] and 48.93% [9] experienced depression. It is evident that there are inconsistencies in the literature investigating the prevalence of anxiety and depression among the UK population during the first COVID‐19 lockdown.

To date, no study has systematically reviewed and meta‐analysed the prevalence of anxiety and depression in the United Kingdom during the first COVID‐19 lockdown. Quantifying the prevalence of anxiety and depression during this time is essential to inform policy development and mental health services which mental health issue requires more intervention efforts to promote positive mental health (Molodynski, McLellan, Craig, & Bhugra, 2020). Furthermore, identifying the prevalence of anxiety and depression during the lockdown allows for comparisons to be made with the corresponding prevalence rates prior to the pandemic. This comparison is paramount to comprehend the severity of the impact of the lockdown on mental health. It is predicted that COVID‐19 will continue to circulate among the global population for years ahead (Torjesen, 2021) urging immediate preparation for the ongoing mental health consequences of the pandemic and potential future lockdowns.

### Study aims

In this study, we will conduct a systematic review and meta‐analysis to determine: (1) the prevalence of anxiety and depression during the first UK COVID‐19 lockdown, (2) how the prevalence of anxiety and depression during the first UK COVID‐19 lockdown compares with pre‐pandemic prevalence rates, and (3) whether the prevalence of anxiety or depression was greater during the first UK COVID‐19 lockdown.

## Method

### Study design

We conducted a systematic review and meta‐analyses to investigate the prevalence of anxiety and depression during the first COVID‐19 lockdown in the United Kingdom.

### Registration and guidelines

Our protocol was pre‐registered with PROSPERO (registration number: [BLINDED]), and we adhered to the preferred reporting items for systematic reviews and meta‐analyses (PRISMA) statement and checklist (see Appendix 1) (Moher, Liberati, Tetzlaff, & Altman, 2009).

### Inclusion and exclusion criteria

A study was included in the review if it investigated the time of the first UK COVID‐19 lockdown (23 March 2020 to 13 May 2020), the sample comprised the UK general population, had a cross‐sectional design and was written in English. Importantly, the studies had to include anxiety and depression prevalence data derived from well‐established psychological assessments with distinct cut‐off scores. Cut‐off scores distinguish non‐clinical from clinical cases of anxiety or depression. While individuals who score below the cut‐off score are judged as non‐clinical, those who score above the cut‐off score are judged as clinical (McHugh & Behar, 2009). Whereas a study was excluded if it assessed a period outside of the first UK COVID‐19 lockdown, the sample consisted of a subpopulation in the United Kingdom, was not cross‐sectional in design, was written in a language other than English, or provided inadequate anxiety and depression prevalence data.

### Search strategy

The literature search strategy was developed by selecting terms and keywords derived from scoping search and expertise in the subject field. The key search terms included (1) COVID‐19, Coronavirus, 2019‐nCoV, SARS‐CoV‐2, Lockdown, (2) United Kingdom, UK, U.K., (3) Anxiety, Anxious, Worry, Stress, Distress, and (4) Depression, Depressive, Depressed, and Sadness (see Appendix 2).

We searched the databases Embase, Medline (PubMed), Web of Science, and PsycINFO for relevant records. The literature search was carried out on 01 September 2021.

### Study selection

Three review authors were involved in the study selection. In case of disagreement about study inclusion and exclusion, consensus was reached by discussion. The studies identified in the literature search were imported to Covidence, a systematic review management tool, where duplicate articles were removed. In the first stage of the study selection, the titles and abstracts were screened against the inclusion and exclusion criteria. Studies that did not appear to meet the inclusion criteria were removed. In the second stage, for studies that appeared to meet the inclusion criteria, the full‐text reports were obtained and examined to decide whether these met the inclusion criteria. Studies that did not meet the inclusion criteria were removed and the reason for exclusion was noted. The studies that met the inclusion criteria entered the third stage of risk of bias assessment.

### Data extraction

One review author performed the data extraction which was confirmed by the other two reviewers after reaching consensus about disagreements by discussion. The data from the final studies were extracted using a pre‐prepared checklist. The items on the checklist included the study’s author, sample size, date range, age range, gender distribution, anxiety and depression psychological assessments with cut‐off scores, and the prevalence of anxiety and depression. Study authors were contacted to obtain missing data, and data that were not provided in a directly usable form were calculated. The data were organized using an Excel spreadsheet to produce a ‘Summary of findings’ table (Table 1). Additionally, the data were qualitatively synthesized in the form of a narrative review to acquire a comprehensive understanding of the characteristics and findings of the studies.

**Table 1:** Summary of findings

					Assessments		Outcomes	
Author	Sample Size	Date range	Age Range	Females (%)	AnxietyCut‐off	Depression Cut‐off	Anxiety (%) (*n*)	Depression (%) (*n*)
Bu et al.	26,720	07.05.20–14.05.20	18–60+	51.00	GAD‐7 ≥10	PHQ‐9 ≥10	19.60 5,237	28.00 7,482
Groarke et al.	1,964	23.03.20–24.04.20	18–87	70.40	GAD‐7 ≥10	PHQ‐9 ≥10	30.30 595	34.01 668
Jacob et al.	902	17.03.20–01.04.20	18–65+	63.80	BAI ≥16	BDI ≥20	30.71 277	18.85 170
Jia et al.	3,097	03.04.20–30.04.20	18–99	84.53	GAD‐7 ≥10	PHQ‐9 ≥10	26.03 806	31.58 978
McPherson et al.	1,958	23.03.20–24.04.20	18–87	69.77	GAD‐7 ≥10	PHQ‐9 ≥10	30.13 590	33.81 662
Neill et al.	1,989	23.03.20–24.04.20	18–87	70.40	GAD‐7 ≥10	PHQ‐9 ≥10	30.30 596	34.00 668
O'Connor et al.	3,077	31.03.20–09.04.20	18–65+	55.10	GAD‐7 ≥10	PHQ‐9 ≥10	21.00 646	26.10 803
Pieh et al.	1,006	21.04.20–01.05.20	18–65+	54.10	GAD‐7 ≥10	PHQ‐9 ≥10	38.97 392	41.15 414
Sharman et al.	1,028	24.04.20–30.04.20	18‐73	72.10	DASS‐21 ≥14	DASS‐21 ≥10	32.30 332	48.93 503
Shevlin et al.	2,025	23.03.20–28.03.20	18–83	51.70	GAD‐7 ≥10	PHQ‐9 ≥10	21.63 438	22.12 448
Taylor et al.	636	04.05.20–09.06.20	18–75	82.23	GAD‐7 ≥10	PHQ‐9 ≥10	31.29 199	35.53 226
White et al.	551^a^ 554^b^	31.03.20–13.04.20	18–76	74.00	HADS ≥8	HADS ≥8	67.51 372	46.57 258
Wilson et al.	887	17.03.20–03.07.20	18–65+	64.04	BAI ≥16	BDI ≥20	31.23 277	19.84 176
Wood et al.	314^a^ 315^b^	01.05.20–02.06.20	18–81	75.24	DASS‐21 ≥14	DASS‐21 ≥10	20.07 65	26.98 85

BAI, Beck Anxiety Inventory; BDI, Beck Depression Inventory; DASS‐21, Depression, Anxiety and Stress Scale ‐ 21 Items; GAD‐7, General Anxiety Disorder ‐ 7 Items; HADS, Hospital Anxiety and Depression Scale; PHQ‐9, Patient Health Questionnaire – 9 Items.

^a^ Anxiety.

^b^ Depression.

### Risk of bias assessment

**Figure 1:**
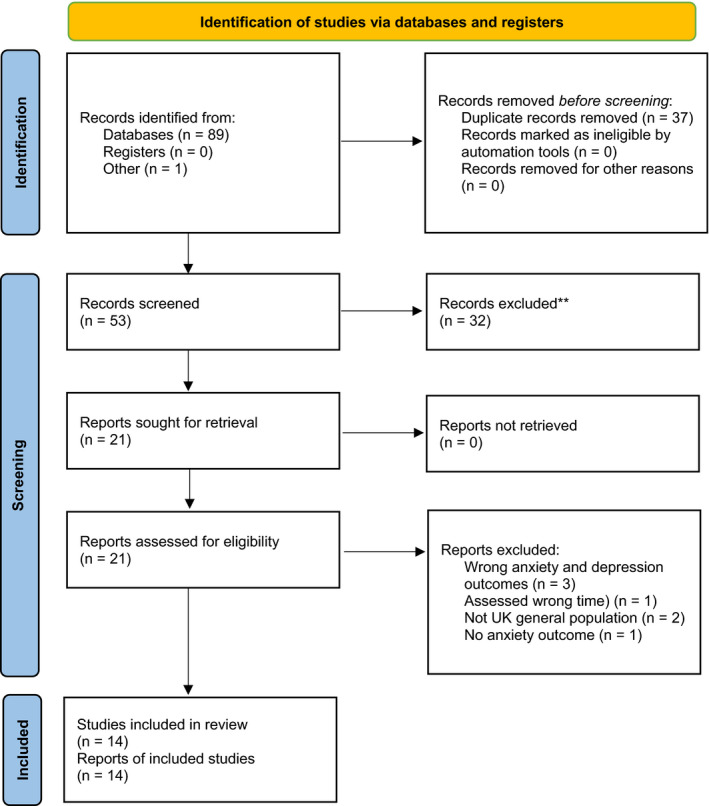
PRISMA flow diagram.

To assess the risk of bias of the studies, the Newcastle‐Ottawa scale (NOS) was used (Wells et al., 2000) (see Appendix 3). The scale consists of five sections, including sample representativeness, sample size, non‐respondents, ascertainment of the outcomes, and quality of statistics reporting. A maximum of 1 point could be awarded per section, hence the total scores range from 0 to 5. Studies were judged to have a low risk of bias (≥3 points) or a high risk of bias (<3 points). If studies were judged to have a high risk of bias, they were excluded. The study selection was recorded in the PRISMA flow diagram (Figure 1).

### Statistical analyses

The meta‐analyses were performed by computing the weighted prevalence (overall effect estimate) of anxiety and depression outcomes with 95% confidence intervals. To assess heterogeneity, the *I* ^2^ (%) test was used (Higgins, Thompson, Deeks, & Altman, 2003). Due to the high heterogeneity observed, random‐effects models were used for both outcomes and the corresponding forest plots were produced.

To visually assess publication bias, funnel plots were produced. To statistically ascertain publication bias, Egger’s test was conducted with a significance level of 0.05 (Egger, Smith, Schneider, & Minder, 1997). Data analysis was performed using the statistical software, Stata (Version 17).

## Results

### PRISMA flow diagram

As shown in the PRISMA flow diagram presented in Figure 1, a total of 90 studies were obtained from the databases and additional sources. After the removal of 37 duplicate studies, the number of studies reduced to 53. In the first stage of study selection, the title and abstract screening, 32 studies were removed resulting in 21 eligible studies. Regarding inter‐rater agreement, the mean Cohen's kappa coefficient was 0.09, indicating slight agreement among the three review authors (Landis & Koch, 1977). In the second stage, the full‐text examination, 7 studies were removed (see Appendix 4), resulting in 14 eligible studies (see Appendix 5). Regarding inter‐rater agreement, the mean Cohen's kappa coefficient was 0.48, indicating moderate agreement between the three review authors (Landis & Koch, 1977).

### Characteristics of studies and participants

Among the 14 articles included in the review, 1 was unpublished (Taylor et al., 2020) and was included to reduce the risk of publication bias. All studies were cross‐sectional in design and used random sampling to recruit participants through online platforms. The studies were conducted between 17 March 20 and 09 June 20 during the time of the first UK COVID‐19 lockdown. The sample sizes ranged from 314 to 26,720 participants who were aged 18 to 90 years old. Females made up 51.00% to 75.25% of the samples. To assess anxiety and depression, two studies used the BAI and BDI (Jacob et al., 2020; Wilson et al., 2021), two studies used the DASS‐21 (Sharman, Roberts, Bowden‐Jones, & Strang, 2021; Wood, Barton, & Smyth, 2021), nine studies used the GAD‐7 and PHQ‐9 (Bu et al., 2021; Groarke et al., 2020; Jia et al., 2020; McPherson, McAloney‐Kocaman, McGlinchey, Faeth, & Armour, 2021; Neill, Blair, Best, McGlinchey, & Armour, 2020; O'Connor et al., 2020; Pieh et al., 2021; Shevlin et al., 2020; Taylor et al., 2020), and one study used the HADS (White & Van Der Boor, 2020b). In the 14 studies included in the review, the prevalence of anxiety (above the clinical cut‐off) ranged from 19.60% to 67.51%. Whereas the prevalence of depression (above the clinical cut‐off) ranged from 18.85% to 47.93%. Of the 14 studies, 3 studies (Jacob et al., 2020; White & Van Der Boor, 2020a; Wilson et al., 2021) demonstrated that the prevalence of anxiety was greater than the prevalence of depression. The other 11 studies demonstrated that the prevalence of depression was greater than the prevalence of anxiety (Bu et al., 2021; Groarke et al., 2020; Jia et al., 2020; McPherson et al., 2021; Neill et al., 2020; O'Connor et al., 2020; Pieh et al., 2021; Sharman et al., 2021; Shevlin et al., 2020; Taylor et al., 2020; Wood et al., 2021).

### Risk of bias assessment

All included studies obtained a final score ranging from 3 to 5 as assessed by the NOS and were, therefore, judged to have a low risk of bias (Wells et al., 2000) (see Appendix 3). Numerous studies did not receive a point for sample representativeness as a consequence of gender bias, specifically, an overrepresentation of females, in the samples.

### Investigating heterogeneity and publication bias

To investigate the heterogeneity of the studies, the *I* ^2^ (%) indices for the prevalence of anxiety (*I* ^2^ = 98.80%) (Figure 5) and depression (*I* ^2^ = 97.70%) (Figure 6) were obtained (Higgins et al., 2003). A reason for the high heterogeneity may be that the samples of the included studies represent a random sample from the larger UK population. Due to the high heterogeneity, random‐effects models were used in the analysis of the findings.

**Figure 2:**
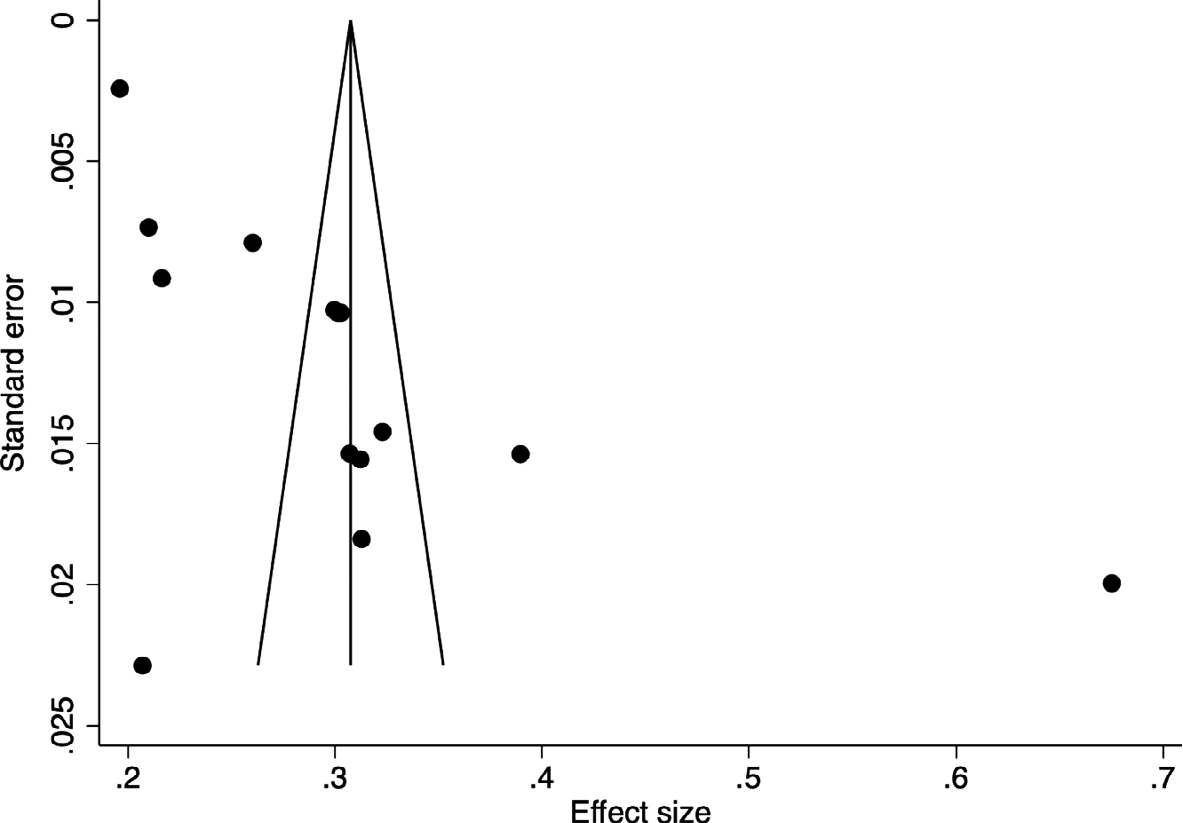
Funnel plot of the prevalence of anxiety during the first UK COVID‐19 lockdown.

**Figure 3:**
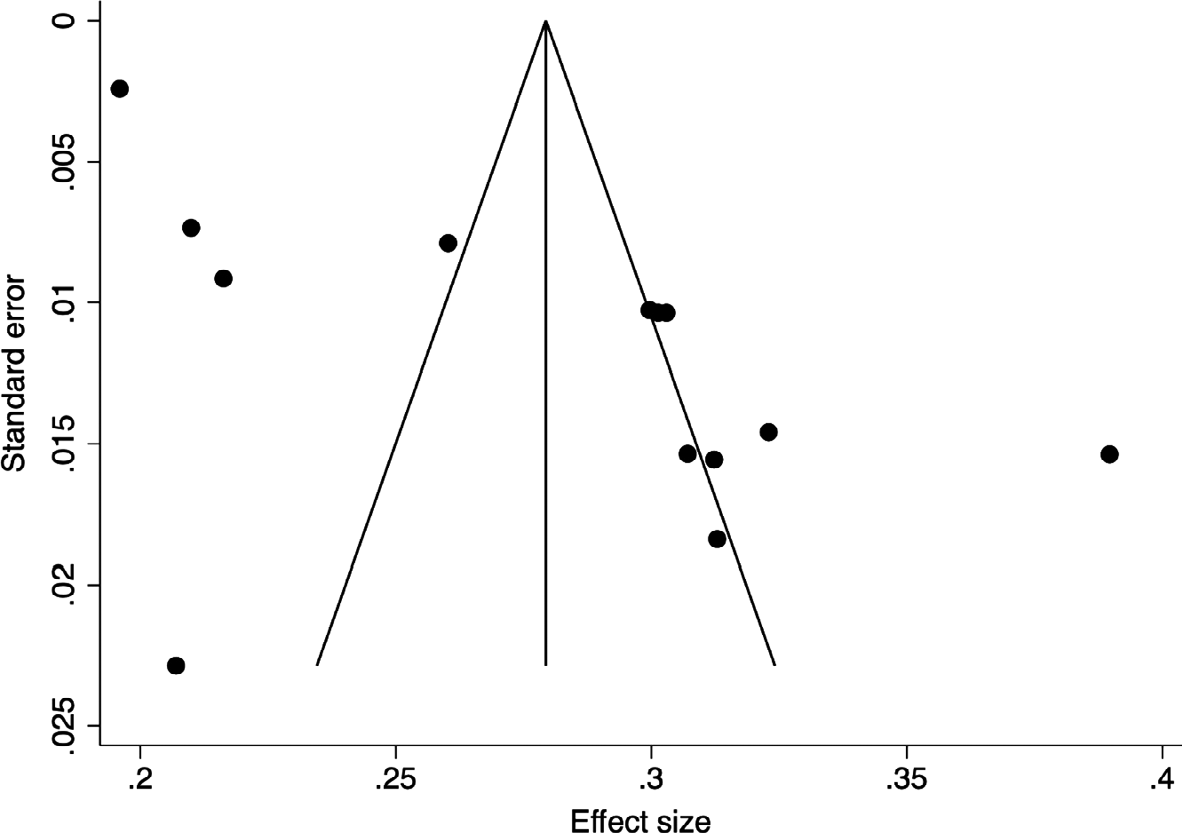
Funnel plot of the prevalence of anxiety during the first UK COVID‐19 lockdown without the small study.

**Figure 4:**
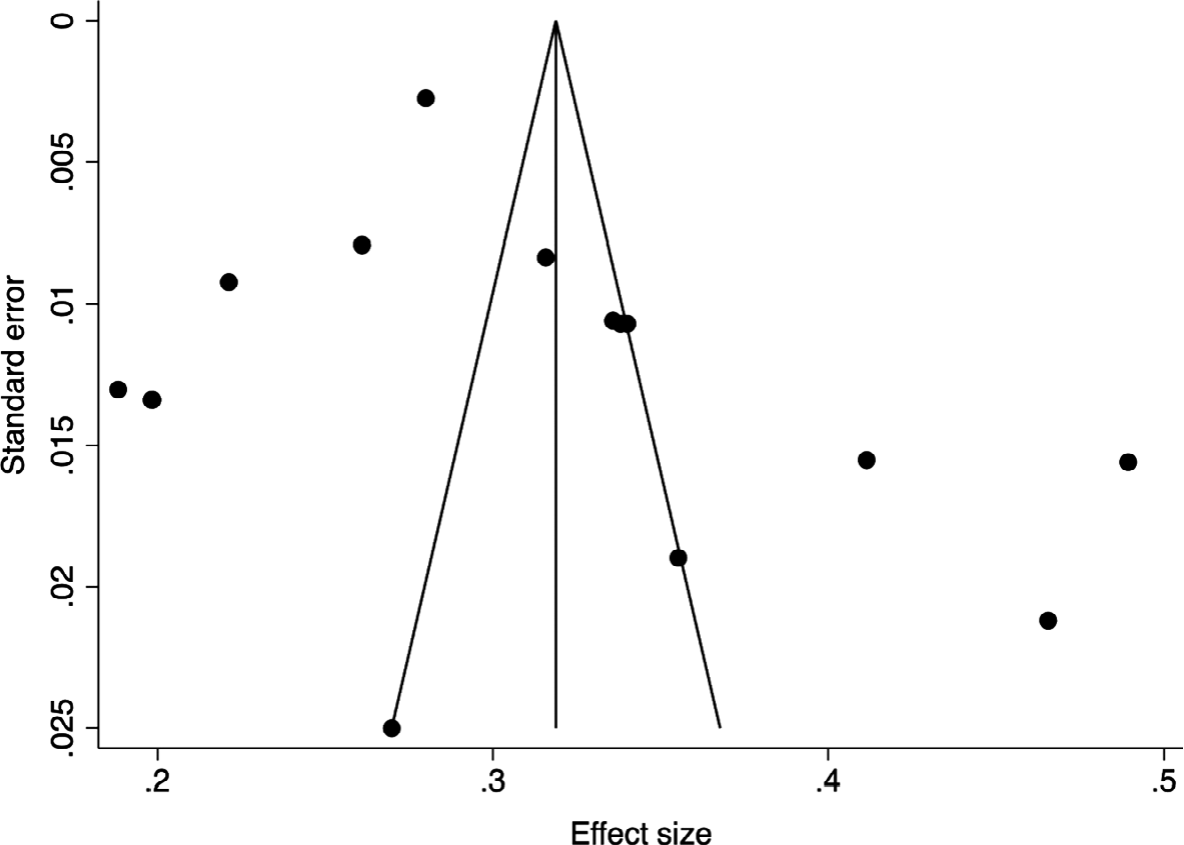
Funnel plot of the prevalence of depression during the first UK COVID‐19 lockdown.

To examine publication bias in the collected articles, funnel plots were created, and the Egger’s test indices were obtained (Egger et al., 1997). The funnel plot of the prevalence of anxiety (Figure 2) illustrates asymmetry, indicating probable publication bias. The indices for the prevalence of anxiety (*p* = .05) further indicate that publication bias is significant. However, it is likely that one small study (White & Van Der Boor, 2020b) influenced the funnel plot symmetry and Egger’s test indices because when the study is removed, the funnel plot is symmetric (Figure 3), and the Egger’s test indices (*p* = .12) indicate that publication bias is not significant. However, the funnel plot of the prevalence of depression (Figure 4) illustrated symmetry, indicating improbable publication bias. The indices for depression (*p* = .20) further indicate that publication bias is not significant.

### Meta‐analyses

**Figure 5:**
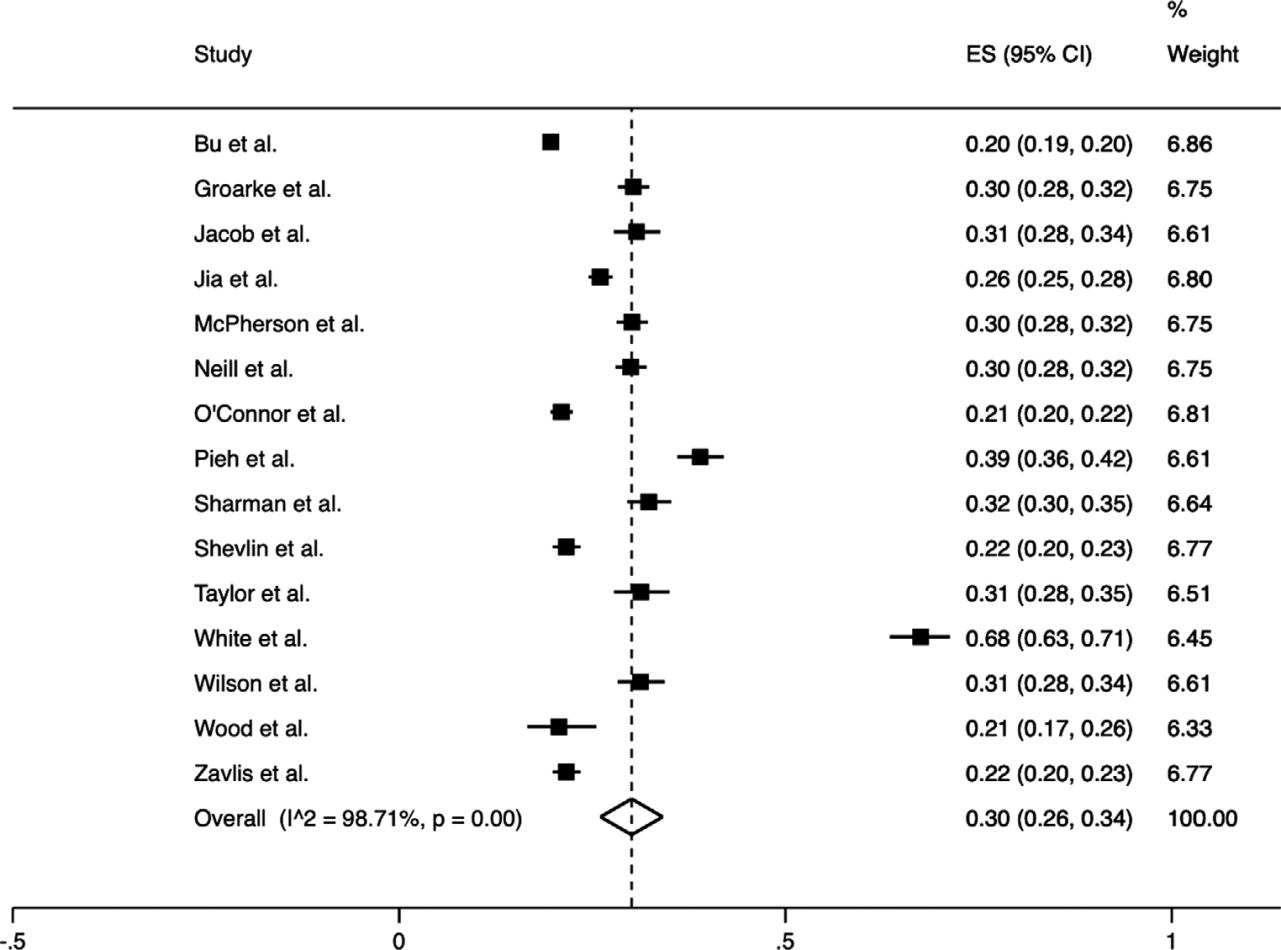
Forest plot of the pooled prevalence of anxiety and its 95% confidence interval during the first UK lockdown (estimates were derived from a random‐effects model).

**Figure 6:**
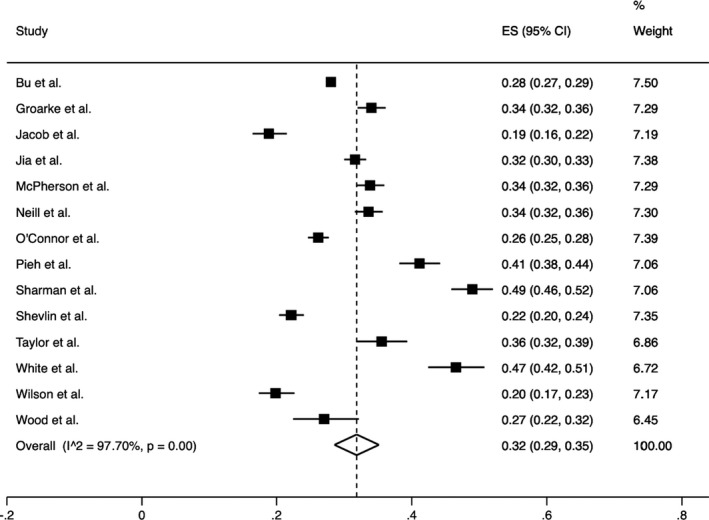
Forest plot of the pooled prevalence of depression and its 95% confidence interval during the first UK lockdown (estimates were derived from a random‐effects model).

The pooled prevalence of anxiety from 14 studies and across 46,154 participants was 31.00% (95% CI = 26.00–35.00) (Figure 5). The pooled prevalence of depression from 14 studies and across 46,158 participants was 32.00% (95% CI = 29.00–35.00) (Figure 6).

## Discussion

### Findings and hypotheses

This work is the first systematic review and meta‐analyses on the prevalence of anxiety and depression during the first COVID‐19 lockdown in the United Kingdom. The review followed the appropriate methods of a systematic review and meta‐analysis for pooling estimates across 15 cross‐sectional studies using clinically valid measures to assess anxiety and depression. The risk of bias in these studies was deemed to be low according to assessment using the NOS scale (Wells et al., 2000) (see Appendix 3).

According to our meta‐analysis, the pooled prevalence of anxiety (above the clinical cut‐off) was 31.00% and the prevalence of depression was 32.00%, the confidence intervals overlapped suggesting that there was no strong evidence for a difference between anxiety and depression prevalence. According to the Global Burden of Disease (GBD) study conducted in 2017, the prevalence of anxiety was 4.65% among the UK general population (Global Burden of Disease Study 2017 Results, 2018). In comparison to the current anxiety prevalence rate of 31.00%, we can estimate that there was a 26.35% increase in the prevalence of anxiety since the commencement of the pandemic. Similarly, according to the GBD study, the prevalence of depression was 4.12% (Global Burden of Disease Study 2017 Results, 2018). In comparison to the current depression prevalence rate of 32.00%, we can estimate that there was a 27.88% increase in the prevalence of depression since the emergence of the pandemic.

### Literature comparison

The prevalence rate of anxiety and depression during the first COVID‐19 lockdown in the United Kingdom aligns with the corresponding global prevalence rate during the pandemic. Castaldelli‐Maia, Marziali, Lu, and Martins (2020) identified a global prevalence rate of 21.30% for anxiety and 24.00% for depression, whereas Salari et al. (2020) determined a global prevalence rate of 31.90% for anxiety and 33.70% for depression. On the global scale, the current prevalence rate of 31.00% for anxiety and 32.00% for depression lies between the prevalence rates obtained by the global reviews indicating that the prevalence of anxiety during the pandemic in the United Kingdom does not deviate from other countries in the world. Similarly, the current UK prevalence rate of anxiety aligns with the corresponding European prevalence rates during the pandemic. While Castaldelli‐Maia et al. (2020) identified a European prevalence rate of 21.90% for anxiety and 26.00% for depression, Salari et al. (2020) determined a European prevalence rate of 23.80% for anxiety and 32.40% for depression. On the European scale, the current anxiety prevalence rate (31.00%) was greater than the prevalence rates obtained by the discussed reviews, indicating that the prevalence of anxiety during the pandemic in the United Kingdom is above average in comparison to other countries in Europe. This may be because the current review investigates a specific lockdown, whereas the discussed reviews explored the pandemic in general. Although social distancing restrictions are consistently enforced during the pandemic, lockdowns entail considerably stricter restrictions that could elevate anxiety. On the European scale, the current depression prevalence rate (32.00%) lies between the prevalence rates obtained by the global reviews. In fact, the prevalence rate of depression obtained by Salari et al. (2020) is almost identical to the current UK prevalence rate. This indicates that the prevalence of depression during the pandemic in the United Kingdom does not deviate from other countries in Europe.

The alignment of the current prevalence rates of anxiety and depression with the prevalence rates on the global and European scale indicates that anxiety and depression during the pandemic are significant concerns across the globe regardless of the unique pandemic restrictions that countries enforce. Moreover, the agreement in prevalence rates demonstrates the robustness of the current findings across different countries, populations, and measures. However, it should be taken into consideration that the discussed global reviews did not investigate a specific lockdown but rather the pandemic in general. Although lockdowns take place during the pandemic, they involve considerably stricter restrictions than periods when no lockdown is in place. This implies that the prevalence rates of anxiety and depression obtained by the global reviews would be expected to be lower than the current prevalence rates. Nonetheless, with or without lockdowns, the pandemic has had detrimental effects on the mental health of individuals worldwide.

### Clinical and policy implications

The findings of the current review introduce significant clinical and policy implications that could contribute to fostering positive mental health outcomes. Awareness of the high prevalence of anxiety and depression during the first lockdown can inform policy development that substantial effort, time, and funding of mental health services are required to support those in need (Molodynski et al., 2020). It is crucial that mental health services are made publicly available, and that seeking support is normalized particularly during this crisis (Salaheddin & Mason, 2016). For example, services should advertise their around‐the‐clock availability to offer advice and support on how to look after one’s mental health during the pandemic through online platforms and telephone helplines (Wright & Caudill, 2020). Specific attention should be paid to subgroups with vulnerability to the disease, lower household income, those diagnosed with mental illness, and people living with children as they are the most vulnerable to suffer anxiety and depression (Fancourt, Steptoe, & Bu, 2020).

Similarly, awareness of the prevalence of anxiety and depression in the United Kingdom can contribute to the development of nation‐specific interventions and initiatives. Although there are global interventions and initiatives to support individuals suffering from anxiety and depression, nation‐specific ones are required to support the UK population specifically (Griner & Smith, 2006). Every country differs in its COVID‐19 restrictions and lockdown measures and, in turn, their mental health outcomes. Hence, taking into account the pandemic restrictions unique to the United Kingdom is essential to develop culturally sensitive mental health interventions and initiatives that target the distinct needs of the population (Castaldelli‐Maia et al., 2020). For instance, considering that the prevalence of depression was greater than that of anxiety during the first UK lockdown, online interventions that teach coping mechanisms to deal with depression‐triggering restrictions should be implemented (Pfefferbaum & North, 2020). With the strong likelihood of coronavirus becoming endemic in the human population, it is critical that the nation is prepared for the co‐occurring mental health crisis as a consequence of inevitable lockdowns (Adalja, 2020).

### Strengths

The current review presents numerous strengths. First, the primary strength of the current study lies in its design. This systematic review and meta‐analyses synthesized all empirical evidence based on specific inclusion criteria to explore the prevalence of anxiety and depression during the first COVID‐19 lockdown in the United Kingdom. Combining data across studies with different measures allows for a more robust estimate of the impact of the lockdown on mental health than is possible from a single study (Møller & Myles, 2016). Additionally, the transparency of each stage in the review allows the reader to focus on the merits of each decision made in compiling the information (Smith & Noble, 2016). Second, the literature search is a strength because broad search terms were used to retrieve the literature and hand searching was employed to avoid missing available literature. Moreover, authors were contacted to obtain missing data and data that were not provided in a directly usable form were calculated. Third, the strict inclusion criteria used, such as only including studies using well‐established and reliable psychological instruments, allows for robust conclusions regarding the clinical levels of anxiety and depression in the lockdown. Finally, the novelty of the current review is a strength because it is the first to investigate the effects of the lockdown in the United Kingdom on anxiety and depression.

### Limitations and future research

However, the findings of the current research should be considered in light of their limitations. First, although the included studies utilized valid psychological instruments to assess for anxiety and depression, they merely provide diagnostic indications that should be confirmed by further clinical evaluation to increase the validity of the findings (Spitzer, Kroenke, Williams, & Löwe, 2006). Second, volunteer sampling may have introduced bias, potentially weakening the validity of the findings. Voluntary response bias may have been introduced because individuals who volunteer may display similar characteristics, thus increasing the risk of yielding an unrepresentative sample (Sharma, 2017). Specifically, individuals who suffer from anxiety or depression may be unmotivated to volunteer (Grahek, Shenhav, Musslick, Krebs, & Koster, 2019). Moreover, the majority of the included studies were characterized by gender bias due to an overrepresentation of females, who have been found to experience a greater prevalence of anxiety and depression than males during the first UK lockdown (Shevlin et al., 2020). Therefore, future research should employ sampling methods, such as stratified or systematic sampling, that avoid voluntary response bias and gender bias to strengthen the validity of the findings. Third, the current review is limited because it focuses on the UK general population which does not allow the findings to be generalized to the global population. This means that the current prevalence rates cannot be applied to other countries to understand how lockdown restrictions affect mental health. Hence, future studies should replicate the current analysis with lockdowns occurring in other countries to understand how lockdown restrictions affect mental health. Finally, the indirect comparison of the current prevalence rates with the corresponding pre‐pandemic prevalence rates obtained from a different study sample is a limitation. Comparing two different samples increases individual differences between participants, which could confound the findings (Karwowski & Cuevas, 2003). Thus, future research should directly compare the prevalence rates obtained from the same study sample, for example, by conducting a longitudinal cross‐sectional design. This would avoid individual differences between participants and ensure the reliability of the findings.

The current research could be strengthened by exploring individual differences in sociodemographic factors, such as age and socioeconomic status, which have been shown to affect anxiety and depression during the lockdown (Kwong et al., 2020; Pierce et al., 2020). Furthermore, the research could be strengthened by considering confounding variables, such as substance abuse and domestic abuse, both of which rose significantly during the pandemic (Bhavsar et al., 2021; Panchal et al., 2020). Future research should consider mediating factors that can exacerbate symptoms of anxiety and depression. In terms of anxiety, these include distressing COVD‐19 news and being at risk of unemployment (Godinic, Obrenovic, & Khudaykulov, 2020; Moghanibashi‐Mansourieh, 2020). In terms of depression, these include loneliness and bereavement (Burrell & Selman, 2020; Luchetti et al., 2020). Identifying mediating factors for each disorder could elucidate the current finding that the prevalence of depression is consistently greater than that of anxiety, and simultaneously aid in the development of tailored support. Taking into account individual differences, confounding variables and mediating factors could increase the internal validity of the research (Skelly, Dettori, & Brodt, 2012).

The current findings provide baseline measures for the prevalence of anxiety and depression during the first COVID‐19 lockdown in the United Kingdom. Future research should longitudinally explore the change in the prevalence of anxiety and depression across subsequent lockdowns. While it is plausible that the population has become habituated to the restrictions, it is also plausible that mental health has deteriorated over time. This research is essential to understand how mental health services and policies can improve to provide optimal support to those suffering from anxiety and depression during lockdowns.

### Conclusion

The COVID‐19 pandemic has created a global state of emergency concerning not only physical health but also mental health. According to the current systematic review and meta‐analyses, it can be concluded that the prevalence of anxiety and depression during the first COVID‐19 lockdown in the United Kingdom was significantly higher than pre‐pandemic prevalence rates. These findings can invaluably inform policymakers and mental health services that substantial effort, time, and funding are required to support those in need. Additionally, they can facilitate the development of culturally sensitive mental health interventions and initiatives that target the distinct needs of the population to preserve and improve mental health during these unprecedented times.

## Ethics statement

The study received ethical approval from the University of Bath Psychology Ethics Committee (reference number: UG 20‐098).

## Data Availability

The data that support the findings of this study are available from the corresponding author upon reasonable request.

## Appendix 1 PRISMA checklist

**Table:**

Section and Topic	Item #	Checklist item	Location where item is reported
TITLE			
Title	1	Identify the report as a systematic review.	1
ABSTRACT			
Abstract	2	See the PRISMA 2020 for Abstracts checklist.	Attached
INTRODUCTION			
Rationale	3	Describe the rationale for the review in the context of existing knowledge.	1‐2
Objectives	4	Provide an explicit statement of the objective(s) or question(s) the review addresses.	2
METHODS			
Eligibility criteria	5	Specify the inclusion and exclusion criteria for the review and how studies were grouped for the syntheses.	3
Information sources	6	Specify all databases, registers, websites, organizations, reference lists, and other sources searched or consulted to identify studies. Specify the date when each source was last searched or consulted.	3
Search strategy	7	Present the full search strategies for all databases, registers, and websites, including any filters and limits used.	3, Appendix 2
Selection process	8	Specify the methods used to decide whether a study met the inclusion criteria of the review, including how many reviewers screened each record and each report retrieved, whether they worked independently, and if applicable, details of automation tools used in the process.	4
Data collection process	9	Specify the methods used to collect data from reports, including how many reviewers collected data from each report, whether they worked independently, any processes for obtaining or confirming data from study investigators, and if applicable, details of automation tools used in the process.	4
Data items	10a	List and define all outcomes for which data were sought. Specify whether all results that were compatible with each outcome domain in each study were sought (e.g., for all measures, time points, and analyses), and if not, the methods used to decide which results to collect.	4
	10b	List and define all other variables for which data were sought (e.g., participant and intervention characteristics, and funding sources). Describe any assumptions made about any missing or unclear information.	4
Study risk of bias assessment	11	Specify the methods used to assess risk of bias in the included studies, including details of the tool(s) used, how many reviewers assessed each study, and whether they worked independently, and if applicable, details of automation tools used in the process.	4‐5
Effect measures	12	Specify for each outcome the effect measure(s) (e.g., risk ratio and mean difference) used in the synthesis or presentation of results.	5
Synthesis methods	13a	Describe the processes used to decide which studies were eligible for each synthesis (e.g., tabulating the study intervention characteristics and comparing against the planned groups for each synthesis (item #5)).	4
	13b	Describe any methods required to prepare the data for presentation or synthesis, such as handling of missing summary statistics, or data conversions.	4
	13c	Describe any methods used to tabulate or visually display results of individual studies and syntheses.	5
	13d	Describe any methods used to synthesize results and provide a rationale for the choice(s). If meta‐analysis was performed, describe the model(s), method(s) to identify the presence and extent of statistical heterogeneity, and software package(s) used.	5
	13e	Describe any methods used to explore possible causes of heterogeneity among study results (e.g., subgroup analysis and meta‐regression).	5
	13f	Describe any sensitivity analyses conducted to assess robustness of the synthesized results.	Not applicable
Reporting bias assessment	14	Describe any methods used to assess risk of bias due to missing results in a synthesis (arising from reporting biases).	5
Certainty assessment	15	Describe any methods used to assess certainty (or confidence) in the body of evidence for an outcome.	5
RESULTS			
Study selection	16a	Describe the results of the search and selection process from the number of records identified in the search to the number of studies included in the review, ideally using a flow diagram.	Figure 1
	16b	Cite studies that might appear to meet the inclusion criteria, but which were excluded, and explain why they were excluded.	6, Appendix 4
Study characteristics	17	Cite each included study and present its characteristics.	6‐7, Table 1
Risk of bias in studies	18	Present assessments of risk of bias for each included study.	7, Appendix 3
Results of individual studies	19	For all outcomes, present, for each study: (a) summary statistics for each group (where appropriate) and (b) an effect estimate and its precision (e.g., confidence/credible interval), ideally using structured tables or plots.	Figures 5 and 6
Results of syntheses	20a	For each synthesis, briefly summarise the characteristics and risk of bias among contributing studies.	6‐7
	20b	Present results of all statistical syntheses conducted. If meta‐analysis was done, present for each the summary estimate and its precision (e.g., confidence/credible interval) and measures of statistical heterogeneity. If comparing groups, describe the direction of the effect.	8, Figures 5 and 6
	20c	Present results of all investigations of possible causes of heterogeneity among study results.	7
	20d	Present results of all sensitivity analyses conducted to assess the robustness of the synthesized results.	Not applicable
Reporting biases	21	Present assessments of risk of bias due to missing results (arising from reporting biases) for each synthesis assessed.	7‐8, Figures 2, 3, 4
Certainty of evidence	22	Present assessments of certainty (or confidence) in the body of evidence for each outcome assessed.	8, Figures 5 and 6
DISCUSSION			
Discussion	23a	Provide a general interpretation of the results in the context of other evidence.	8‐10
	23b	Discuss any limitations of the evidence included in the review.	12‐13
	23c	Discuss any limitations of the review processes used.	12‐13
	23d	Discuss implications of the results for practice, policy, and future research.	10‐11, 12‐14
OTHER INFORMATION			
Registration and protocol	24a	Provide registration information for the review, including register name and registration number, or state that the review was not registered.	3
	24b	Indicate where the review protocol can be accessed, or state that a protocol was not prepared.	3
	24c	Describe and explain any amendments to information provided at registration or in the protocol.	Not applicable
Support	25	Describe sources of financial or non‐financial support for the review, and the role of the funders or sponsors in the review.	Attached
Competing interests	26	Declare any competing interests of review authors.	Attached
Availability of data, code, and other materials	27	Report which of the following are publicly available and where they can be found: template data collection forms; data extracted from included studies; data used for all analyses; analytic code; any other materials used in the review.	Submitted

## Appendix 2 Medline (PubMed) search strategy

1. COVID‐19[Title] OR Coronavirus[Title] OR 2019‐ncov[Title] OR SARS‐cov‐2[Title]
2. United Kingdom[Title] OR UK[Title] OR U.K.[Title] AND Lockdown[Title]
3. Anxiety[Title/Abstract] OR Anxious[Title/Abstract] OR Worry[Title/Abstract] OR Stress[Title/Abstract] OR Distress[Title/Abstract]
4. Depression[Title/Abstract] OR Depressive[Title/Abstract] OR Depressed[Title/Abstract] OR Sadness[Title/Abstract]
5. #1 AND #2 AND #3 AND #4

## Appendix 3 Newcastle‐Ottawa scale

**Table:**

Study	Sample representativeness	Sample size	Non‐respondents	Ascertainment of outcomes	Quality of statistics reporting	Total score	Risk of bias
Bu et al.	1	1	1	1	1	5	Low
Groarke et al.	0	1	1	1	1	4	Low
Jacob et al.	0	1	1	1	1	4	Low
Jia et al.	0	1	1	1	1	4	Low
McPherson et al.	0	1	1	1	1	4	Low
Neill et al.	0	1	1	1	1	4	Low
O'Connor et al.	1	1	1	1	1	5	Low
Pieh et al.	1	1	1	1	1	5	Low
Sharman et al.	0	1	1	1	1	4	Low
Shevlin et al.	1	1	1	1	1	5	Low
Taylor et al.	0	1	1	1	1	4	Low
White et al.	0	1	1	1	1	4	Low
Wilson et al.	0	1	1	1	1	4	Low
Wood et al.	0	0	1	1	1	3	Low

*Notes* One point was awarded for the following items. *Sample representativeness*: The population was representative of the general UK population. *Sample size*: The sample size was greater than or equal to 385 participants. *Non‐respondents*: The comparability between respondent and non‐respondent characteristics was established and there was a satisfactory response rate. *Ascertainment of outcomes*: The study employed well‐established psychological assessments with valid cut‐off scores (e.g., GAD‐7 ≥ 10, PHQ‐9 ≥ 10). *Quality of statistics reporting*: The study reported statistics with appropriate measures of the prevalence of anxiety and depression. The item scores were summed to generate a total modified risk of bias score for each study. Total scores range from 0 to 5. Studies were judged to have a low risk of bias (≥3 points) or a high risk of bias (<3 points).

## Appendix 4 References of articles excluded based on examination of full text and reasons for exclusion

**Table:**

Reference (*n* = 7)	Reason for exclusion
Codagnone, C., Bogliacino, F., Gómez, C., Charris, R., Montealegre, F., Liva, G., Lupiáñez‐Villanueva, F., Folkvord, F., & Veltri, G. A. (2020). Assessing concerns for the economic consequence of the COVID‐19 response and mental health problems associated with economic vulnerability and negative economic shock in Italy, Spain, and the United Kingdom. PLoS ONE, 15(10). https://doi.org/10.1371/journal.pone.0240876	Wrong anxiety and depression outcomes.
Dawson, D. L., & Golijani‐Moghaddam, N. (2020). COVID‐19: Psychological flexibility, coping, mental health, and wellbeing in the UK during the pandemic. Journal of Contextual Behavioral Science, 17, 126‐134. https://doi.org/10.1016/j.jcbs.2020.07.010	Assessed wrong time.
Jacob, L., Smith, L., Armstrong, N. C., Yakkundi, A., Barnett, Y., Butler, L., McDermott, D. T., Koyanagi, A., Shin, J. I., Meyer, J., Firth, J., Remes, O., López‐Sánchez, G. F., & Tully, M. A. (2021). Alcohol use and mental health during COVID‐19 lockdown: A cross‐sectional study in a sample of UK adults [Article]. Drug and Alcohol Dependence, 219. https://doi.org/10.1016/j.drugalcdep.2020.108488	Wrong anxiety and depression outcomes.
Kwong, A. S. F., Pearson, R. M., Adams, M. J., Northstone, K., Tilling, K., Smith, D., Fawns‐Ritchie, C., Bould, H., Warne, N., Zammit, S., Gunnell, D. J., Moran, P. A., Micali, N., Reichenberg, A., Hickman, M., Rai, D., Haworth, S., Campbell, A., Altschul, D., Flaig, R., McIntosh, A. M., Lawlor, D. A., Porteous, D., & Timpson, N. J. (2021). Mental health before and during the COVID‐19 pandemic in two longitudinal UK population cohorts. British Journal of Psychiatry, 218(6), 334‐343. https://doi.org/10.1192/bjp.2020.242	Not UK general population.
Mehl, A., Bergey, F., Cawley, C., & Gilsdorf, A. (2020). Syndromic Surveillance Insights from a Symptom Assessment App Before and During COVID‐19 Measures in Germany and the United Kingdom: Results From Repeated Cross‐Sectional Analyses. Journal of Medical Internet Research, 8(10), Article e21364. https://doi.org/10.2196/21364	No anxiety outcome.
Pieh, C., Probst, T., Budimir, S., & Humer, E. (2021). Associations between Relationship Quality and Mental Health during COVID‐19 in the United Kingdom. International Journal of Environmental Research and Public Health, 18(6), Article 2869. https://doi.org/10.3390/ijerph18062869	Not UK general population.
Smith, L., Jacob, L., Yakkundi, A., McDermott, D., Armstrong, N. C., Barnett, Y., López‐Sánchez, G. F., Martin, S., Butler, L., & Tully, M. A. (2020). Correlates of symptoms of anxiety and depression and mental wellbeing associated with COVID‐19: a cross‐sectional study of UK‐based respondents. Psychiatry Research, 291. https://doi.org/10.1016/j.psychres.2020.113138	Wrong anxiety and depression outcomes.

## Appendix 5 References of articles included in the review

**Table:**

Reference (*n* = 14)
Bu, F., Mak, H. W., & Fancourt, D. (2021). Rates and predictors of uptake of mental health support during the COVID‐19 pandemic: an analysis of 26,720 adults in the UK in lockdown. Social Psychiatry and Psychiatric Epidemiology. https://doi.org/10.1007/s00127‐021‐02105‐w
Groarke, J. M., Berry, E., Graham‐Wisener, L., McKenna‐Plumley, P. E., McGlinchey, E., & Armour, C. (2020). Loneliness in the UK during the COVID‐19 pandemic: Cross‐sectional results from the COVID‐19 Psychological Wellbeing Study. PLoS ONE, 15(9), Article e0239698. https://doi.org/10.1371/journal.pone.0239698
Jacob, L., Tully, M. A., Barnett, Y., Lopez‐Sanchez, G. F., Butler, L., Schuch, F., López‐Bueno, R., McDermott, D., Firth, J., Grabovac, I., Yakkundi, A., Armstrong, N., Young, T., & Smith, L. (2020). The relationship between physical activity and mental health in a sample of the UK public: A cross‐sectional study during the implementation of COVID‐19 social distancing measures. Mental Health and Physical Activity, 19. https://doi.org/10.1016/j.mhpa.2020.100345
Jia, R., Ayling, K., Chalder, T., Massey, A., Broadbent, E., Coupland, C., & Vedhara, K. (2020). Mental health in the UK during the COVID‐19 pandemic: cross‐sectional analyses from a community cohort study. British Medical Journal Open, 10(9), Article e040620. https://doi.org/10.1136/bmjopen‐2020‐040620
McPherson, K. E., McAloney‐Kocaman, K., McGlinchey, E., Faeth, P., & Armour, C. (2021). Longitudinal analysis of the UK COVID‐19 Psychological Wellbeing Study: Trajectories of anxiety, depression and COVID‐19‐related stress symptomology. Psychiatry Research, 304, 114138‐114138. https://doi.org/10.1016/j.psychres.2021.114138
Neill, R., Blair, C., Best, P., McGlinchey, E., & Armour, C. (2020). Media Consumption and Mental Health during COVID‐19 lockdown: A UK Cross‐sectional study across England, Wales, Scotland and Northern Ireland. Journal of Public Health. https://doi.org/10.1007/s10389‐021‐01506‐0
O'Connor, R. C., Wetherall, K., Cleare, S., McClelland, H., Melson, A. J., Niedzwiedz, C. L., O'Carroll, R. E., O'Connor, D. B., Platt, S., Scowcroft, E., Watson, B., Zortea, T., Ferguson, E., & Robb, K. A. (2021). Mental health and well‐being during the COVID‐19 pandemic: longitudinal analyses of adults in the UK COVID‐19 Mental Health & Wellbeing study. British Journal of Psychiatry, 218(6), 326‐333, Article Pii s0007125020002123. https://doi.org/10.1192/bjp.2020.212
Pieh, C., Budimir, S., Delgadillo, J., Barkham, M., Fontaine, J. R. J., & Probst, T. (2021). Mental Health during COVID‐19 Lockdown in the United Kingdom. Psychosomatic Medicine, 83(4), 328‐337. https://doi.org/10.1097/PSY.0000000000000871
Sharman, S., Roberts, A., Bowden‐Jones, H., & Strang, J. (2021). Gambling in COVID‐19 Lockdown in the UK: Depression, Stress, and Anxiety [Article]. Frontiers in Psychiatry, 12. https://doi.org/10.3389/fpsyt.2021.621497
Shevlin, M., McBride, O., Murphy, J., Miller, J. G., Hartman, T. K., Levita, L., Mason, L., Martinez, A. P., McKay, R., Stocks, T. V. A., Bennett, K. M., Hyland, P., Karatzias, T., & Bentall, R. P. (2020). Anxiety, depression, traumatic stress and COVID‐19‐related anxiety in the UK general population during the COVID‐19 pandemic. British Journal of Psychiatry Open, 6. https://doi.org/10.1192/bjo.2020.109
Taylor, G., Adams, S., Craft, S., Bekteshi, V., Sillero‐Rejon, C., Tamayo, R. M., Bartlett, J., & Freeman, T. (2020). Impact of COVID‐19 social restrictions on lifestyle and wellbeing: a cross‐country longitudinal cohort study with three waves [Unpublished raw data].
White, R. G., & Van Der Boor, C. (2020). Impact of the COVID‐19 pandemic and initial period of lockdown on the mental health and well‐being of adults in the UK. British Journal of Psychiatry Open, 6(5), Article e90. https://doi.org/10.1192/bjo.2020.79
Wilson, J. J., McMullan, I., Blackburn, N. E., Klempel, N., Yakkundi, A., Armstrong, N. C., Brolly, C., Butler, L. T., Barnett, Y., Jacob, L., Koyanagi, A., Smith, L., & Tully, M. A. (2021). Changes in dietary fat intake and associations with mental health in a UK public sample during the COVID‐19 pandemic. Journal of Public Health. https://doi.org/10.1093/pubmed/fdab009
Wood, C. J., Barton, J., & Smyth, N. (2021). A cross‐sectional study of physical activity behaviour and associations with wellbeing during the UK coronavirus lockdown. Journal of Health Psychology, Article 1359105321999710. https://doi.org/10.1177/1359105321999710
